# Anti-Cancer Effects of Xanthones from Pericarps of Mangosteen

**DOI:** 10.3390/ijms9030355

**Published:** 2008-03-14

**Authors:** Yukihiro Akao, Yoshihito Nakagawa, Munekazu Iinuma, Yoshinori Nozawa

**Affiliations:** Gifu International Institute of Biotechnology, 1-1 Naka-Fudogaoka, Kakamigahara, Gifu 504-0838, Japan; Tel: +81-583-71-4646, Fax: +81-583-71-4412

**Keywords:** anti-cancer effect, Xanthones, apoptosis, α-mangostin

## Abstract

Mangosteen, *Garcinia mangostana* Linn, is a tree found in South East Asia, and its pericarps have been used as traditional medicine. Phytochemical studies have shown that they contain a variety of secondary metabolites, such as oxygenated and prenylated xanthones. Recent studies revealed that these xanthones exhibited a variety of biological activities containing anti-inflammatory, anti-bacterial, and anti-cancer effects. We previously investigated the anti-proliferative effects of four prenylated xanthones from the pericarps; α-mangostin, β-mangostin, γ-mangostin, and methoxy-β-mangostin in various human cancer cells. These xanthones are different in the number of hydroxyl and methoxy groups. Except for methoxy-β-mangostin, the other three xanthones strongly inhibited cell growth at low concentrations from 5 to 20 μM in human colon cancer DLD-1 cells. Our recent study focused on the mechanism of α-mangostin-induced growth inhibition in DLD-1 cells. It was shown that the anti-proliferative effects of the xanthones were associated with cell-cycle arrest by affecting the expression of cyclins, cdc2, and p27; G1 arrest by α-mangostin and β-Mangostin, and S arrest by γ-mangostin. α-Mangostin found to induce apoptosis through the activation of intrinsic pathway following the down-regulation of signaling cascades involving MAP kinases and the serine/threonine kinase Akt. Synergistic effects by the combined treatment of α-mangostin and anti-cancer drug 5-FU was to be noted. α-Mangostin was found to have a cancer preventive effect in rat carcinogenesis bioassay and the extract from pericarps, which contains mainly α-mangostin and γ-mangostin, exhibited an enhancement of NK cell activity in a mouse model. These findings could provide a relevant basis for the development of xanthones as an agent for cancer prevention and the combination therapy with anti-cancer drugs.

## 1. Overview

**Figure 1.**
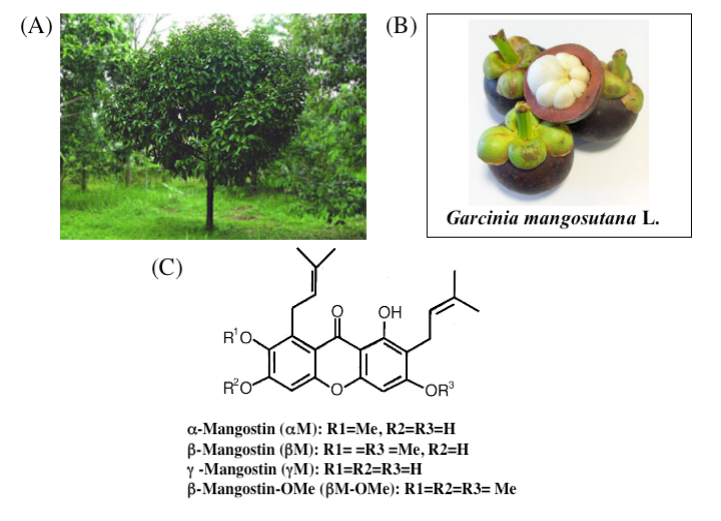
The *Garcinia mangostana* Linn tree (A), the appearance of mangosteen fruit (B) and the chemical structures of xanthones included in the pericarps (C).

The mangosteen tree has been cultivated for centuries in tropical areas of the world. The tree is presumed to have originated in Southeast Asia or Indonesia and has largely remained indigenous to Malay Peninsula, Myanmar, Thailand, Cambodia, Vietnam and the Moluccas (Figure 1A). The white, inner pulp of the mangosteen fruit is highly praised as one of the best tasting of all tropical fruits. The scientific name is *Garcinia mangostana*. The entire fruit is typically 2.5–7.5 cm in diameter, roughly the size of a tangerine (Figure 1B). The rind (or skin) of the fruit is 0.6–1.0 cm thick and contains a purplish pigment. The inner pulp consists of four to eight juicy, white-colored segments (fruit portion, Figure 1B). The edible portion of the fruit comprises only about 25% of the total volume, whereas the remainder is tough, bitter pericarp which exudes a yellow resin (hence the term xanthones or yellow in Greek)(Figure 1B). The mangosteen rind, leaves and bark have been used as folk medicine for thousands of years. The thick mangosteen rind has been and is used for treating catarrh, cystitis, diarrhea, dysentery, eczema, fever, intestinal ailments, pruritis and other skin ailments. The mangosteen leaves are also used by some natives in teas and for diarrhea, dysentery, fever, and thrush. It is also known that concentrates of mangosteen bark can be used for genito-urinary afflictions and stomatosis.

## 2. Introduction

Increasing attention has been paid to primitive medicinal plants and dietary factors to search for new substances with potentially effective anti-cancer activity. A large number of natural products have been evaluated as potential chemopreventive or therapeutic agents. In fact, among these compounds, paclitaxel, etoposide, camptothecin, and vincristine, have been used as anticancer drugs. Epidemiological studies have shown that dietary phytochemicals provide beneficial effects on cancer prevention [1–4]. In this context, evidence-based biofactors for cancer prevention are strongly required for practical use. Among them, polyphenols are of great interest as chemopreventive agents because of their anti-oxidative and possible anti-cancer activity [1–6].

In our series of investigations to search for anti-cancer agents from plant sources, all the polyphenols and terpenoids tested which exhibited an anti-proliferative effect, were observed to induce apoptosis by targeting mitochondria with a decreased membrane potential, leading to the activation of the intrinsic apoptotic signal transduction [7–13]. In some cases, the early responsive signaling cascades including protein kinases MAPK and Akt referring to growth and survival, respectively, were down-regulated [13].

Our previous reports indicated a potent anti-proliferative activity of 4 xanthones (α-mangostin, β-mangostin, γ-mangostin, and methoxy- β-mangostin) from the pericarps of mangosteen against human leukemia HL60 cells. Interestingly, α-mangostin was observed to induce mitochondrial dysfunction [11]. Moreover, it induced cell-cycle arrest and apoptosis in human colon cancer DLD-1 cells [14]. In this review, we discuss the mechanism of anti-cancer effect of xanthones and the possibility of chemopreventive agents for cancer, especially in α-mangostin and γ-mangostin.

## 3. Chemistry of Xanthones

The subsurface chemistry of the mangosteen pericarp comprises an array of polyphenolic acids including xanthones and tannins that assure astringency to discourage infestation by insects, fungi, plant viruses, bacteria and animal predation while the fruit is immature. Color changes and softening of the pericarp are natural processes of ripening, which indicates that the fruit can be eaten and the seeds finish developing. Among the constituents of the pericarps, xanthones are biologically active phenols that naturally occur in a restricted group of plants [15–17]. Over 200 xanthones are currently known to exist in nature and approximately 50 of them are found in the mangosteen. The xanthones possess a six-carbon conjugated ring structure with multiple double carbon bonds. The chemical structures of 4 major xanthones contained in percarps are shown in Figure 1C. The prenyl group is considered to be implicated in the internalization into the cell, which in turn leads to interaction with the signal transduction molecules and the proteins involved in mitochondria permeability transition [18,19].

## 4. Growth Inhibitory Effect of Prenylated Xanthones

**Figure 2.**
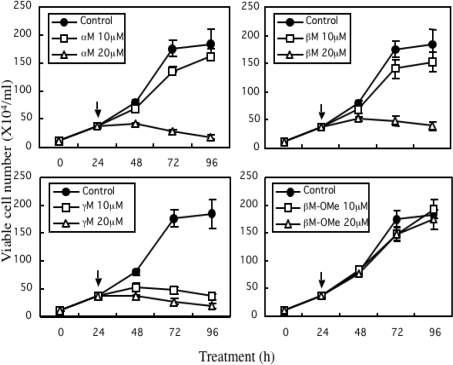
Effect of xanthones on cell growth in human colon cancer DLD-1 cells.

**Figure 3.**
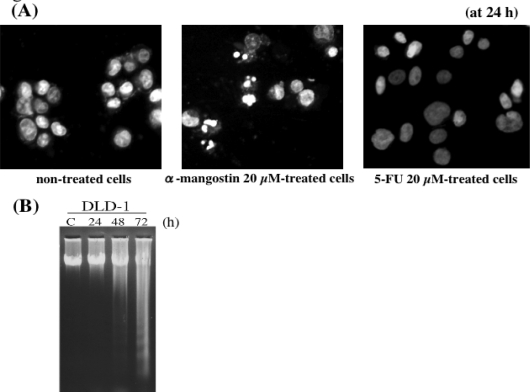
The cell death induced by α-mangostin and 5-FU. Hoechst 33342 staining (A) and nucleosomal DNA fragmentation (B).

The major 4 structurally similar prenylated xanthones [α-mangostin (αM), β-mangostin (βM), γ-mangostin (γM), and methoxy-β-mangostin (βM–ME)] from the pericarps of mangosteen wereexamined for the effect on the growth of human colon cancer DLD-1 cells (Figure 2). Except formethoxy-β-mangostin, other xanthones displayed growth inhibitory effects. From the values of the IC_50_, the inhibitory activity was estimated; βM-ME<βM<αM<γM (Table 1). The Hoechst 33342 staining and DNA electrophoretic analysis demonstrated that the anti-proliferative effect of α-mangostin, which is the major constituent of the extract, is due to the apoptotic process (Figure 3).

In comparison with the anti-cancer drugs such as 5-FU, actinomycin D and camptothecin used for the patients with colon cancer, the IC_50_ of α-mangostin was close to that of 5-FU (Table 1). However, the morphological changes by α-mangostin was quite distinct from those induced by 5-FU (Figure 3A).

## 5. Mechanism of α-Mangostin-inducing Apoptosis

**Figure 4.**
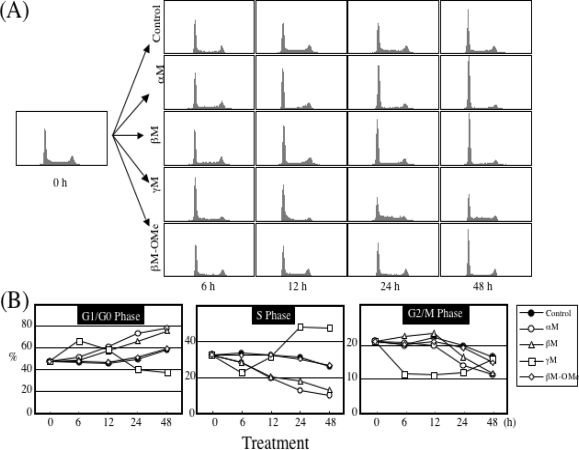

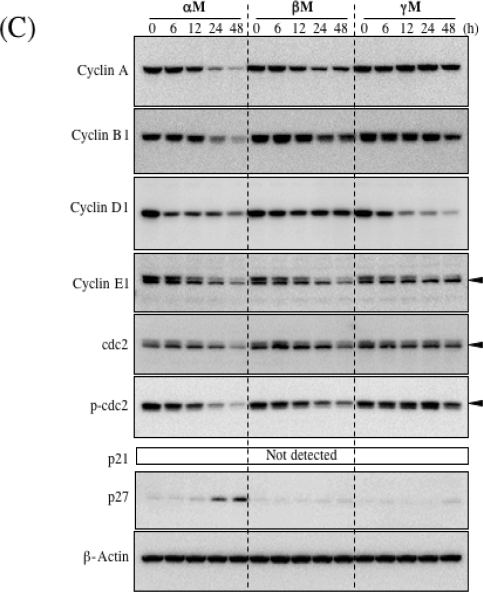
Effect of xanthones on cell cycle progression.

**Figure 5.**
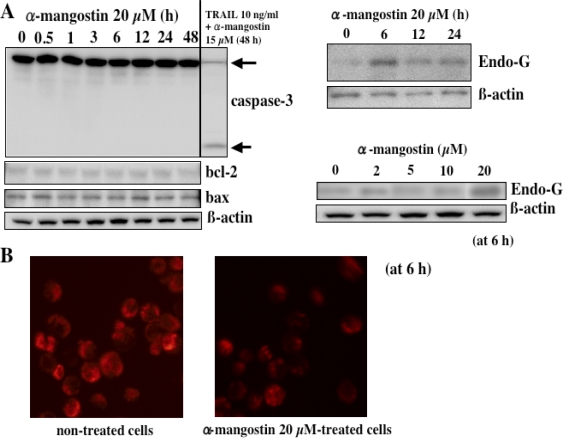
α-Mangostin-induced apoptosis in DLD-1 cells.

In our previous study, it was demonstrated that α-mangostin activated caspase-9 and -3 but not -8 in HL60 cells, indicating that α-mangostin may mediate the mitochondrial pathway in the apoptotic process [11]. Parameters of mitochondrial dysfunctions such as swelling, loss of membrane potential, decrease in intracellular ATP, ROS accumulation, and cytochrome c/AIF release, were observed within 1 or 2 h after the treatment, indicating that α-mangostin preferentially targets mitochondria in the early phase [11]. Interestingly, replacement of hydroxyl group by methoxy group (Figure 1C) remarkably decreased the potency to cause mitochondrial dysfunction. It was also shown that the cytotoxicity was correlated with the decrease in the mitochondrial membrane potential. Furthermore, we demonstrated that α-mangostin induced a cell cycle arrest at G1/S and the subsequent apoptosis via the intrinsic pathway in DLD-1 cells, while a cell cycle arrest by γ-mangostin was at S phase (Figure 4A and B) [14]. The changes in expression of cell cycle regulatory proteins were shown in Figure 4C. α-Mangostin-induced apoptosis was mediated by a caspase-independent pathway via mitochondria with the release of Endo-G (Figure 5) [13]. Endo-G, a known 30-kD nuclease residing in mitochondria, is able to induce nucleosomal DNA fragmentation [13].

Many serine/threonine protein kinases control cell growth, proliferation, differentiation, cell cycle, survival and death. Mitogen-activated protein kinases (MAPKs) and Akt kinase are key regulatory proteins in cells. MAPKs are a widely conserved family of serine/threonine protein kinases involved in many cellular processes such as cell proliferation, differentiation, motility, and death [20]. Akt, another serine/threonine protein kinase, is associated with cell survival, growth, and glycogen metabolism [21]. Various phytochemicals, including epigallocatechin-3-gallate [22], resveratrol [23], arucanolide [10] etc., have been shown to modulate the signaling pathways of MAPKs and/or Akt, leading to growth inhibition and cell death.

**Figure 6.**
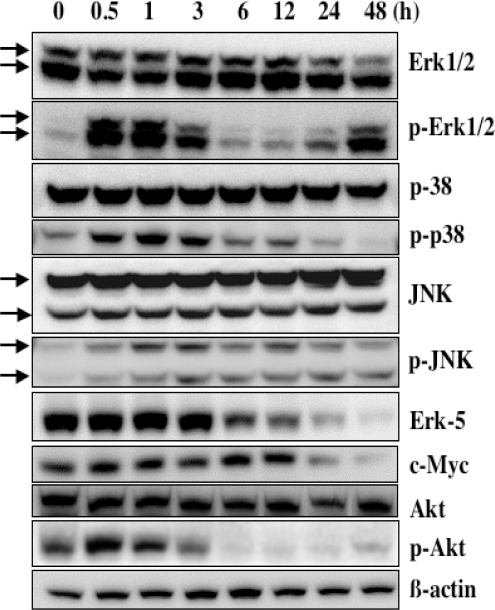
Activities of MAP kinases and Akt kinase in 20 μM α-mangostin-treated DLD-1 cells.

The levels of phosphorylation of p38 and p-JNK appeared to change within 24 h after the treatment with α-mangostin, but their changes could not be properly explained (Figure 6). The levels of p-Erk1/2 showed 2 peaks at the early and late phases. Recently, the dual expression of p-Erk1/2 was also 2 peaks observed in HT-22 cells exposed to glutamate-induced oxidative stress [24]. Erk1/2 may play a dual role, acting first as a cellular adaptive response at the initial phase and then as a cytotoxic response at the later stage. As reported [24], the decline in p-Erk1/2 after the later peak may be associated with the apoptotic machinery. On the other hand, in the Akt signaling the level of p-Akt was markedly reduced at 6 h following α-mangostin treatment (Figure 6), coincident with the occurrence of apoptosis. Therefore, down-regulation of Akt signaling could participate in the mechanism of apoptosis induced by α-mangostin.

**Figure 7.**
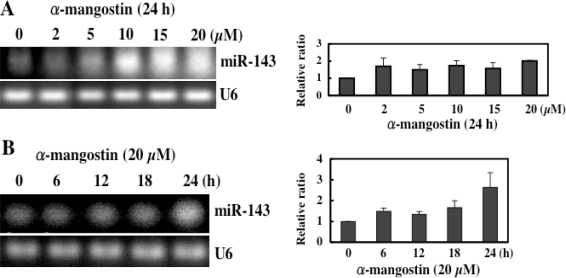
Semi-qRT-PCR-evaluated or TaqMan^®^ probe assay (Real-Time PCR)-evaluated miRNA-143.

**Figure 8.**
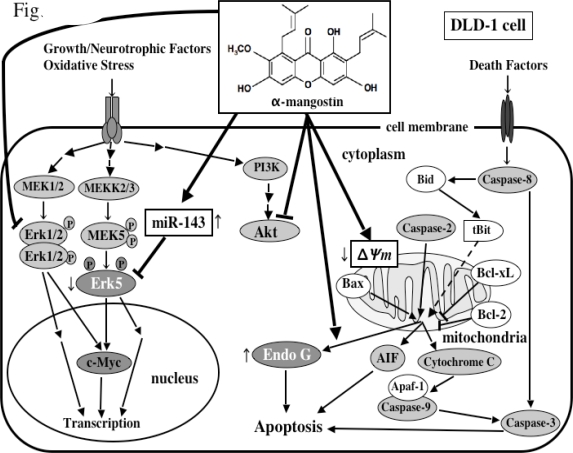
A scheme showing the possible mechanisms of α-mangostin-induced cell death.

Intriguingly, we have recently found that α-mangostin up-regulated the expression of miRNA-143 (Figure 7)[13]. miR-143 is highly expressed especially in normal colon tissues, but its expression in human colon cancer tumors is markedly decreased [25,26]. We determined its target mRNA to be *ERK5* by introducing miRNA-143 into DLD-1 cells [25,26]. α-Mangostin increased the expression levels of miRNA-143 in the process of the apoptotic cell death probably by modulating its transcription and/or the upstream signals associated with the transcription factors of miR-143 [13]. The molecular mechanism of the apoptotic cell death induced by α-mangostin in DLD-1 cells is schematically summarized in Figure 8. α-Mangostin first affects the cell cycle i.e. arrest at G1/S and thereafter induces apoptosis which is mediated by the intrinsic pathway through mitochondria, which follows the modulation of the growth-related signal transduction via MAPK Erk1/2 and Akt, and the expression level of miRNA-143, a target of *ERK5*.

## 6. Combined Treatment of α-Mangostin with Anticancer Drugs

In view of recent phytochemical studies, it has been pointed out that such substances included in vegetables and fruits could affect the efficacy of anti-cancer drugs and their metabolism [27], because many of the patients with cancer take folk medicines and supplements in addition to anti-cancer drugs. Therefore, it is important to study the interaction between phytochemicals and anti-cancer drugs. Furthermore, strategies aimed at enhancing the therapeutic efficiency of anti-cancer drugs and decreasing the side effect involve its administration schedule and also its use in combination with phytochemicals for a better treatment response [27,28]. 5-FU, which is one of the most effective chemotherapeutic agents for colorectal adenocarcinoma [28], can produce response rates of ∼11% when used as a single agent [28]. For example, folinic acid [28], leucovorin (LV) [29–31], oxaliplatin (L-OHP), LV in the FOLFOX regimen [32], and irinotecan (CPT-11) and LV in the FOLFIRI regimen [33] are combination therapies for colorectal cancer patients.

**Figure 9.**
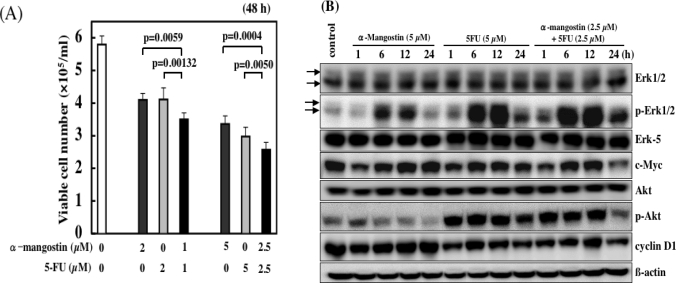
Synergistic growth-inhibiting effect in the combined treatment with α-mangostin and 5-FU in DLD-1 cells.

We demonstrated the synergistic effect on cell growth when 5-FU was used with α-mangostin (total 2 and 5 μM) (Figure 9A). The growth inhibition by 5-FU was probably due to cell cycle arrest at the concentrations tested [34], because no apoptotic cells were observed (Figure 3A). At more than 15 μM α-mangostin, apoptotic cells were observed, whereas at lower concentrations α-mangostin most likely causes cell cycle arrest like 5-FU. Therefore, the synergistic effect by the combined treatment at the total 2 and 5 μM concentrations was probably due to the additional enhancement of the machinery leading to cell cycle arrest. Indeed, the expression of cell cycle-related proteins such as cyclin D1 and c-Myc at total 5 μM was significantly reduced at 24 h, compared with that found in single each agent (Figure 9B).

It is possible that the mechanism of growth suppression by α-mangostin is different from that of 5-FU at more than 15 μM, because the growth inhibition obtained by a single treatment with α-mangostin was greater compared with that by the combined treatment. It is possible that the more potent apoptosis-inducing activity of α-mangostin which was observed at more than 15 μM, was not induced by the combination with 5-FU at both 7.5 μM and 10 μM. In this context, the activation of MAPKs and Akt signal pathways, which were changed by the treatment with 20 μM α-mangostin alone, could be reduced in the single treatment of α-mangostin or 5-FU. Thus, phytochemicals are conceivable to exert a considerable effect on the efficacy of anti-cancer agents, depending on their concentrations, by modulating the intracellular signaling pathways [27]. The enhanced efficacy of α-mangostin with other anti-cancer agents was also shown by our recent study.

## 7. Cancer Preventive Effect *in vivo*

**Figure 10.**
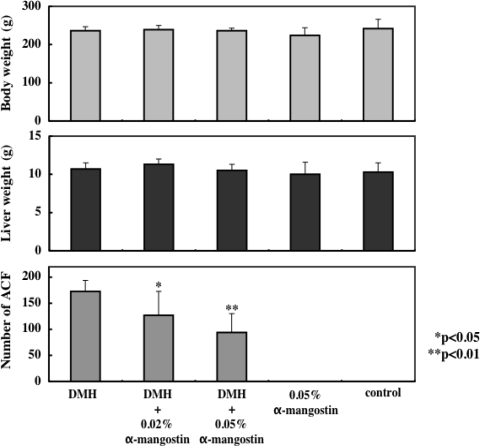
Body weight, liver weight, and the number of atypical crypt foci in the colon from BALB/C control mice and mice treated with 0.02% and 0.05% dietary α-mangostin.

We examined whether α-mangostin has short-term chemopreventive effects on putative preneoplastic lesions involved in rat colon carcinogenesis [35]. Rats in groups 1–3 were given a subcutaneous injection of carcinogen 1,2-dimethylhydrazine (DMH)(40 mg/kg body weight) once a week for 2 weeks. Dietary administration of α-mangostin at doses of 0.02% and 0.05% α-mangostin significantly inhibited the induction and/or development of aberrant crypt foci (ACF) (P<0.05 for 0.02% α-mangostin, P<0.01 for 0.05% α-mangostin), when compared to the DMH-treated group (group 1)(Figure 10). Moreover, treatment of rats with 0.05% α-mangostin significantly decreased dysplastic foci and β-catenin accumulated crypts, to below the group 1 values [35]. The finding that α-mangostin has potent chemopreventive effects in our short-term colon carcinogenesis bioassay system suggests that the longer exposure would result in suppression of tumor development.

## 8. Immunomodulatory Effect

Natural killer (NK) cell works as the main immune cells of the innate immunity, and it is especially important in the eradication of the tumor cells and the virus infected cells. However, the activity of the NK cell decreases with aging after the peak at about 15 years old. The morbidity rate of people with cancer is rising by aging, which may be related to the decline of the activity of the NK cell activity.

**Figure 11.**
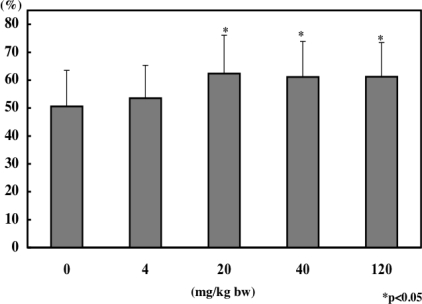
Effect of mangosteen pericarps extract Panaxanthone on the activity of NK cells in mice.

We investigated the effect of α-mangostin on NK cell activity using a mouse model. We administrated the α-mangostin-enriched extract from pericarps named Panaxanthone (α-mangostin, 80–90%; γ-mangostin, 5–10%) to mice everyday by gavage with different doses for 30 days. The NK cell activity was determined by measuring LDH after the incubating of YAC-1 cells (target cells) and splenocytes (effector cells) at the ratio of 1:50. The activities of 20 and 40 mg/kg groups were significantly elevated compared with that in control group 0 mg/kg (Figure 11). A significant increase in the NK cell activity by Panaxanthone was also observed in the human pilot study using healthy people at the dose of 150 mg/day per a person for 7 days.

## 9. Conclusion and Future Perspectives

The anti-proliferative activity of α-mangostin is markedly high, because the IC_50_ of α-mangostin is almost same as that of 5-FU in DLD-1 cells. Its activity is mainly due to apoptosis. The apoptotic observations such as morphological changes and DNA ladder formation emerged at 24 h-treatment with α-mangostin, while the decrease in the mitochondrial membrane potential and the release of Endo-G observed at 6 h-treatment. These events were preceded by the inactivation of the signaling cascades involving Erk1/2 and Akt at 3 h-treatment. The cell cycle regulatory proteins cyclin D1 and cdc2 were also down-regulated at 3 h-treatment. Since the swelling of mitochondria was observed at 1 h-treatment, α-mangostin most likely attacks the proteins involved in permeability transition of mitochondria. This event could trigger the cell cycle arrest and apoptosis (Figure 8).

Considering the chemopreventive effects of phytochemicals, they may depend on three main activities: anti-oxidant, apoptosis inducing, and phase II enzymes inducing. In our studies, we have verified such activities in α-mangostin. It has been already known that the ORAC (Oxygen Radical Absorbance Capacity) value of mangosteen is makedly high [36](www.naturalproductsassoc.org). α-Mangostin increased the expression of glutathione S-transferase (GST) at 0.5–5.0 μM for 12 h-treatment in human hepatocellular carcinoma HuH-7 cells. Furthermore, recent study has revealed that the suppression of inflammatory reaction by phytochemicals leads to cancer prevention. The xanthones, which have anti-oxidant activity, have been reported to reduce the expression of cyclooxygenase-2 (COX-2) [37–39] and to suppress the nuclear factor-κB (NF-κB)[37,40].

Recently, we succeeded in crystallizing the extract from the pericarps and found that the substance contains more than 90% xanthones (α-mangostin, 80–90%; γ-mangostin, 5–10%). This was named Panaxanthone, and assessed to be safe by a conventional safety test using mouse model. The safety was further confirmed by the fact that these xanthones have been used as a folk medicine for many years and more than 160 kinds of fruits juice containing whole extract of mangosteen are distributed worldwide. The safety clinical trial of Panaxanthone is under progress, leading to development for a cancer preventive and therapeutic agent.

## Figures and Tables

**Table 1.** Growth inhibitory effect (IC50) of α-mangostin and anti-cancer drugs in DLD-1 cells.

agent	IC_50_
α-mangostin	7.5 μM
β-managostin	8.1 μM
γ-managostin	7.1 μM
5-FU	4.5 μM
Actinomycin D	0.34 μM
Camptothecin	7.0 nM
Taxol	5.0 nM
Etoposide	2.7 nM

The starting number of cells was 1×10^5^/ml.
